# The Complete Chloroplast Genome of Ye-Xing-Ba (*Scrophularia dentata*; Scrophulariaceae), an Alpine Tibetan Herb

**DOI:** 10.1371/journal.pone.0158488

**Published:** 2016-07-08

**Authors:** Lianghong Ni, Zhili Zhao, Gaawe Dorje, Mi Ma

**Affiliations:** 1 School of Pharmacy, Shanghai University of Traditional Chinese Medicine, Shanghai, China; 2 Tibetan Traditional Medical College, Lhasa, China; Chinese Academy of Medical Sciences, Peking Union Medical College, CHINA

## Abstract

*Scrophularia dentata* is an important Tibetan medicinal plant and traditionally used for the treatment of exanthema and fever in Traditional Tibetan Medicine (TTM). However, there is little sequence and genomic information available for *S*. *dentata*. In this paper, we report the complete chloroplast genome sequence of *S*. *dentata* and it is the first sequenced member of the Sect. Tomiophyllum within *Scrophularia* (Scrophulariaceae). The gene order and organization of the chloroplast genome of *S*. *dentata* are similar to other Lamiales chloroplast genomes. The plastome is 152,553 bp in length and includes a pair of inverted repeats (IRs) of 25,523 bp that separate a large single copy (LSC) region of 84,058 bp and a small single copy (SSC) region of 17,449 bp. It has 38.0% GC content and includes 114 unique genes, of which 80 are protein-coding, 30 are transfer RNA, and 4 are ribosomal RNA. Also, it contains 21 forward repeats, 19 palindrome repeats and 41 simple sequence repeats (SSRs). The repeats and SSRs within *S*. *dentata* were compared with those of *S*. *takesimensis* and present certain discrepancies. The chloroplast genome of *S*. *dentata* was compared with other five publicly available Lamiales genomes from different families. All the coding regions and non-coding regions (introns and intergenic spacers) within the six chloroplast genomes have been extracted and analysed. Furthermore, the genome divergent hotspot regions were identified. Our studies could provide basic data for the alpine medicinal species conservation and molecular phylogenetic researches of Scrophulariaceae and Lamiales.

## Introduction

Ye-Xing-Ba (Tibetan name), a common Tibetan herb, is traditionally used for the treatment of exanthema and fever in Traditional Tibetan Medicine (TTM). As a medicinal alpine species [1], *Scrophularia dentata* Royle ex Benth. (Scrophulariaceae) is one of the original plants of the Tibetan herb [2]. In recent years, our research team has been concentrating on the ethnobotanical survey of Tibetan herbs since 1992 [3, 4]. Based on our specimen collection and taxonomic identification, some chemical and pharmacological studies on *S*. *dentata* have been conducted by our team [5, 6, 7]. Because of the harsh living environment and increasing demand for medicinal use of the alpine plant, it is of utmost urgency to develop a conservation strategy for the species. Also, selection of excellent germplasm requires more efficient genetic data and molecular markers [8]. However, there is little sequence and genomic information available for the species in the GenBank, except for nrDNA ITS sequences, some chloroplast and mitochondrion segment sequences submitted by our team (http://www.ncbi.nlm.nih.gov/nuccore/?term=Scrophularia+dentata).

Chloroplast contains its own independent genome encoding a specific set of proteins [9]. The plastid genomes typically are composed of a large single copy (LSC) region and a small single copy (SSC) region, which are separated by two copies of inverted repeats (IRs) [10]. The small size of the chloroplast genome, about 115 to 165 kb in length, makes it suitable for complete sequencing and the data can be further applied to DNA bar coding and phylogeny construction [11, 12]. As complete chloroplast genome sequences contain sufficient information, sequencing of whole chloroplast genomes is essential for the analyses of plant species [13, 14].

*S*. *dentata* belongs to Scrophulariaceae, which is a large family of Lamiales, consisting of over 3,000 species [15]. Also, the genus *Scrophularia* has ca. 200 species and is divided into two sections, i.e. Sect. Scrophularia and Sect. Tomiophyllum [16]. However, the complete chloroplast genome of *Scrophularia takesimensis* within Sect. Scrophularia is the first published whole sequence in *Scrophularia*. This is the first sequenced member of the Sect. Tomiophyllum in the paper. Details of the *S*. *dentata* chloroplast genome structure and organization are reported and compared with previously annotated chloroplast genomes of other Lamiales species. Our studies could expand understanding of the diversity of *Scrophularia* cp genomes and provide basic data for the alpine medicinal species conservation and molecular phylogenetic researches of Scrophulariaceae and Lamiales.

## Materials and Methods

### Plant material

Samples of *S*. *dentata* were collected in Lhasa, Tibet, China. The voucher specimens were deposited in the herbarium of Shanghai University of Traditional Chinese Medicine (field number: XZ201416). Also, the location of the specimens is not within any protected area.

### DNA extraction, genome sequencing and validation

Total chloroplast DNA was extracted from 100 g of fresh leaves using a sucrose gradient centrifugation method improved by Li et al. [17]. The genome was sequenced and assembled on an Illumina MiSeq platform following Gogniashvili et al. [18]. The four junctions between the SSC/LSC and IRs were validated by PCR amplification and Sanger sequencing, and nine other fragments were selected to validate the genome sequence further (S1 Table) [19].

### Genome annotation

The chloroplast genome was annotated using the annotator DOGMA [20], coupled with manual corrections. For all of the tRNA genes, the annotations were verified using tRNAscan-SE1.21 [21]. The genome map was drawn by GenomeVX [22].

### Genome comparison

The complete cp genomes of *S*. *dentata* and five other Lamiales species were compared using the mVISTA program [23]. The boundaries between IR and SC regions of these species were also compared and analysed.

### Repeat structure

The REPuter program [24] was used to identify repeats (forward, palindrome, complement and reverse sequences). The size and identity of the repeats were limited to no less than 30 bp and 90%, respectively, with Hamming distance equal to 3 [25, 26]. Meanwhile, simple sequence repeats (SSRs) were detected using MISA [27] by setting the minimum number of repeats to 10, 5, 4, 3, 3 and 3 for mono-, di-, tri-, tetra-, penta- and hexanucleotides. The repeats and SSRs of *S*. *dentata* were compared with those of *S*. *takesimensis*, the only one complete chloroplast genome available in Scrophulariaceae.

## Results and Discussion

### Genome organization and features

The junction regions between IRs and SSC/LSC and nine additional regions were confirmed by PCR amplification and Sanger sequencing. We compared these sequences to the assembled genome and no mismatch or indel was observed, which validated the accuracy of genome sequencing and assembly. The chloroplast genome sequence of *S*. *dentata* has been submitted to GenBank (accession number: KT428154).

**Fig 1:**
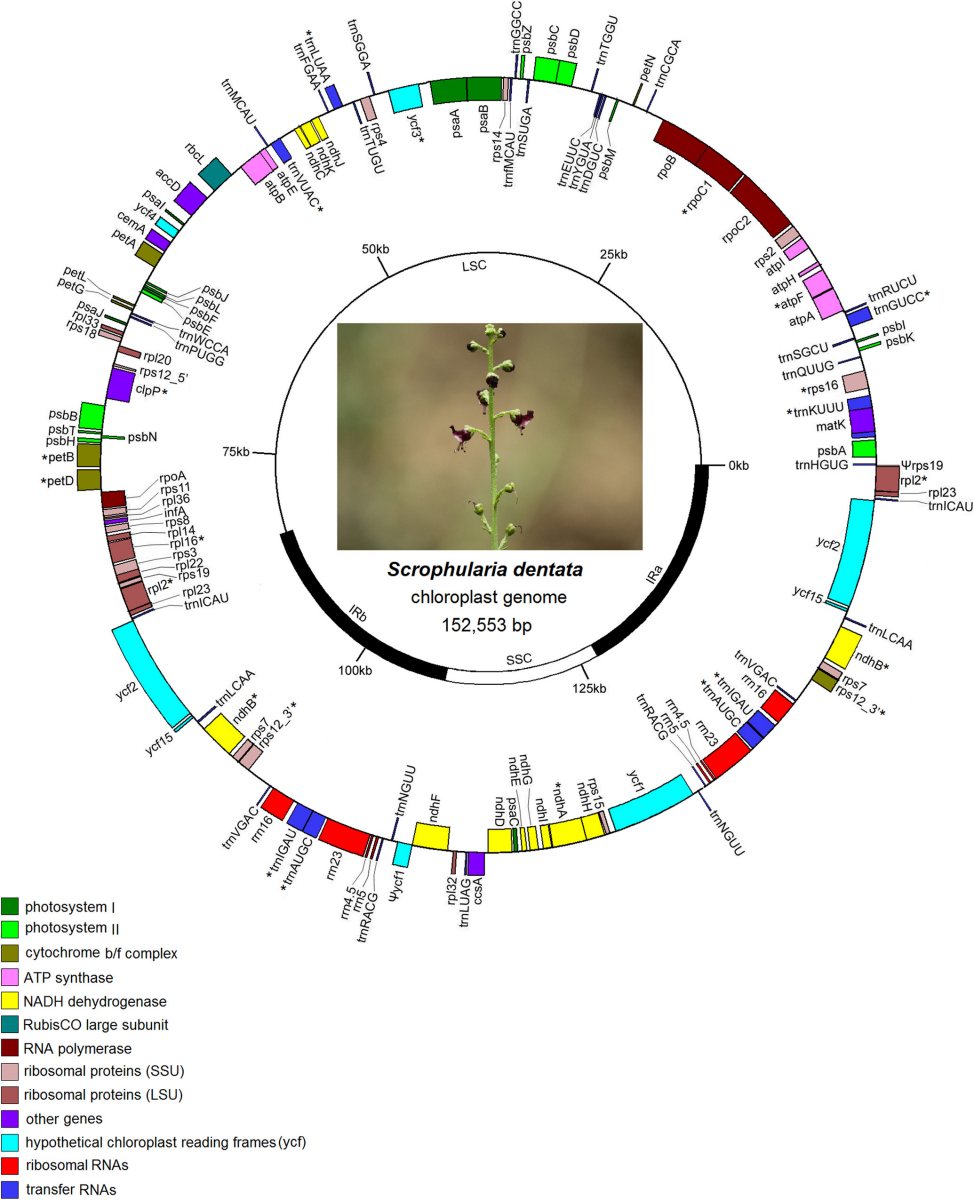
Chloroplast genome map of *Scrophularia dentata*. Genes drawn inside the circle are transcribed clockwise, and those outside are counterclockwise. Genes belonging to different functional groups are shown in different colors. The asterisks indicate genes which contain intron(s).

The complete chloroplast genome of *S*. *dentata* has a total length of 152,553 bp, with a pair of inverted repeats (IRs) of 25,523 bp that separate a large single copy (LSC) region of 84,058 bp and a small single copy (SSC) region of 17,449 bp (Fig 1). The total GC content is 38.0%, which is similar to the published asterids cp genomes [28, 29]. And the GC content is unevenly distributed in the genome. The GC content of IRs (43.1%) is higher than that of LSC and SSC region (36.0 and 32.29%). The high GC content in the IR regions is due to the increased presence of GC nucleotides in the four rRNA genes: *rrn5*, *rrn4*.*5*, *rrn23* and *rrn16*, which is congruent to what has been found in other chloroplast genomes [30, 31].

The chloroplast genome of *S*. *dentata* encodes a total of 114 unique genes, of which 18 are duplicated in IR regions. Among the 114 genes, there are 80 protein-coding genes (70.2%), 30 transfer genes (26.3%) and 4 rRNA genes (3.5%) (Table 1). 18 genes contain introns, 15 (nine protein-coding and six tRNA genes) of which contain one intron and three (*clpP*, *ycf3* and *rps12*) contain two introns (Table 2). The *rps12* gene is a trans-spliced gene, with the first exon located in LSC region and the other two exons duplicated in IR regions.

**Table 1:** Genes present within *Scrophularia dentata* chloroplast genome.

	Group of genes	Gene names
1	Photosystem I	*psaA*, *psaB*, *psaC*, *psaI*, *psaJ*
2	Photosystem II	*psbA*, *psbB*, *psbC*, *psbD*, *psbE*, *psbF*, *psbH*, *psbI*, *psbJ*, *psbK*, *psbL*, *psbM*, *psbN*, *psbT*, *psbZ*
3	Cytochrome b/f complex	*petA*, petB*, petD*, *petG*, *petL*, *petN*
4	ATP synthase	*atpA*, *atpB*, *atpE*, atpF*, *atpH*, *atpI*
5	NADH dehydrogenase	ndhA*, ndhB*(×2), *ndhC*, *ndhD*, *ndhE*, *ndhF*, *ndhG*, *ndhH*, *ndhI*, *ndhJ*, *ndhK*
6	RubisCO large subunit	*rbcL*
7	RNA polymerase	*rpoA*, *rpoB*, rpoC1*, *rpoC2*
8	Ribosomal proteins (SSU)	*rps2*, *rps3*, *rps4*, *rps7*(×2), *rps8*, *rps11*, rps12**(×2), *rps14*, *rps15*, *rps16*, *rps18*, *rps19*
9	Ribosomal proteins (LSU)	rpl2*(×2), *rpl14*, rpl16*, *rpl20*, *rpl22*, *rpl23*(×2), *rpl32*, *rpl33*, *rpl36*
10	Other genes	clpP**, *matK*, *accD*, *ccsA*, *infA*, *cemA*
11	Proteins of unknown function	*ycf1*, *ycf2*(×2), ycf3**, *ycf4*, *ycf15*(×2)
12	Ribosomal RNAs	*rrn4*.*5*(×2), *rrn5*(×2), *rrn16*(×2), *rrn23*(×2)
13	Transfer RNAs	trnA-UGC*(×2), *trnC-GCA*, *trnD-GUC*, *trnE-UUC*, *trnF-GAA*, *trnG-GCC*, trnG-UCC*, *trnH-GUG*, *trnI-CAU*(×2), trnI-GAU*(×2), trnK-UUU*, *trnL-CAA*(×2), trnL-UAA*, *trnL-UAG*, *trnfM-CAU*, *trnM-CAU*, *trnN-GUU*(×2), *trnP-UGG*, *trnQ-UUG*, *trnR-ACG*(×2), *trnR-UCU*, *trnS-GCU*, *trnS-GGA*, *trnS-UGA*, *trnT-GGU*, *trnT-UGU*, *trnV-GAC*(×2), trnV-UAC*, *trnW-CCA*, *trnY-GUA*

*Gene containing one intron.

**Gene containing two introns.

(×2) Two gene copies in the IRs.

**Table 2:** Genes with introns within *Scrophularia dentata* chloroplast genome and the length of exons and introns.

Gene	Location	Exon I (bp)	Intron I (bp)	Exon II (bp)	Intron II (bp)	Exon III (bp)
*petB*	LSC	6	720	642		
*petD*	LSC	8	735	475		
*atpF*	LSC	144	723	411		
*ndhA*	SSC	552	1067	531		
*ndhB*	IR	777	679	756		
*rpoC1*	LSC	435	756	1626		
rps12*	LSC	114	-	232	537	26
*rps16*	LSC	42	848	225		
*rpl2*	IR	393	664	435		
*rpl16*	LSC	9	886	399		
*clpP*	LSC	69	727	291	626	228
*ycf3*	LSC	129	702	228	720	153
*trnA-UGC*	IR	38	813	35		
*trnG-UCC*	LSC	23	683	47		
*trnI-GAU*	IR	42	943	35		
*trnK-UUU*	LSC	37	2517	35		
*trnL-UAA*	LSC	37	488	50		
*trnV-UAC*	LSC	38	585	37		

* The *rps12* is a trans-spliced gene with the 5' end located in the LSC region and the duplicated 3' end in the IR regions.

Protein-coding regions account for 59.6% of the whole genome, while tRNA and rRNA regions account for 4.6% and 5.9%, respectively. The remaining regions are noncoding sequences, including intergenic spacers, introns and pseudogenes. There are two pseudogenes identified: *ycf1* and *rps19*. They are located in the boundary regions between IRb/SSC and IRa/LSC, respectively. The lack of their protein-coding ability is due to the partial gene duplication.

### Comparison to other Lamiales species

The chloroplast genome of *S*. *dentata* was compared with other five publicly available Lamiales genomes from different families, i.e., *Boea hygrometrica* (Gesneriaceae), *Hesperelaea palmeri* (Oleaceae), *Salvia miltiorrhiza* (Lamiaceae), *Andrographis paniculata* (Acanthaceae) and *Sesamum indicum* (Pedaliaceae) (Table 3). Organization of the Lamiales chloroplast genome is conserved; neither translocations nor inversions were detected in the analyses. However, there are differences in terms of genome size. The *A*. *paniculata* chloroplast genome is the shortest (150,249 bp), while that of *H*. *palmeri* (155,820 bp) is longer than other species. The genome size variation is attributed mainly to the difference in the length of the LSC region, similar to the features in Asteraceae chloroplast genomes [36]. Also, the average size of the six Lamiales chloroplast genomes is 152,794 bp.

**Table 3:** Comparison of the base composition of six Lamiales chloroplast genomes.

	*Scrophularia dentata*	*Boea hygrometrica*	*Hesperelaea palmeri*	*Salvia miltiorrhiza*	*Andrographis paniculata*	*Sesamum indicum*
LSC	84,058 bp	84,692 bp	86,616 bp	82,695 bp	82,459 bp	85,170 bp
SSC	17,449 bp	17,901 bp	17,780 bp	17,555 bp	17,190 bp	17,872 bp
IR	25,523 bp	25,450 bp	25,712 bp	25,539 bp	25,300 bp	25,141 bp
Total	152,553 bp	153,493 bp	155,820	151,328 bp	150,249 bp	153,324 bp
CG content	38.0%	37.6%	37.8%	36.2%	38.3%	38.2%
Coding gene	80	86	80	80	80	80
tRNA	30	29	30	30	30	30
rRNA	4	4	4	4	4	4
References	this study	[32]	[33]	[34]	NC_022451	[35]

**Fig 2:**
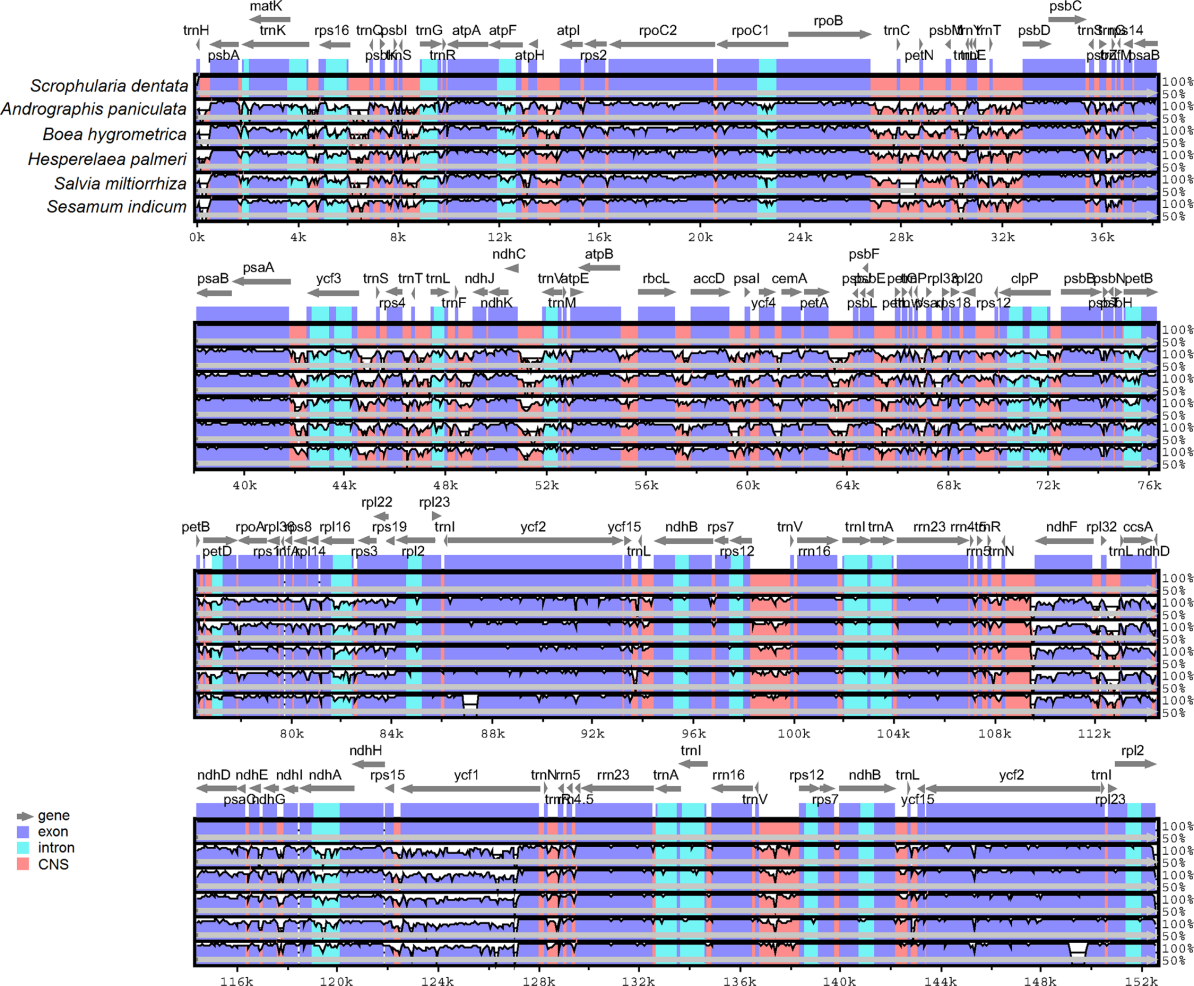
Sequence alignment of six chloroplast genomes in the Lamiales order using mVISTA program with *S*. *dentata* as reference. The top line shows genes in order (transcriptional direction indicated with arrow). A cut-off of 70% identity was used for the plots, and the Y-scale represents the percent identity between 50–100%. Genome regions are color-coded as exon, intron, and conserved non-coding sequences (CNS).

**Fig 3:**
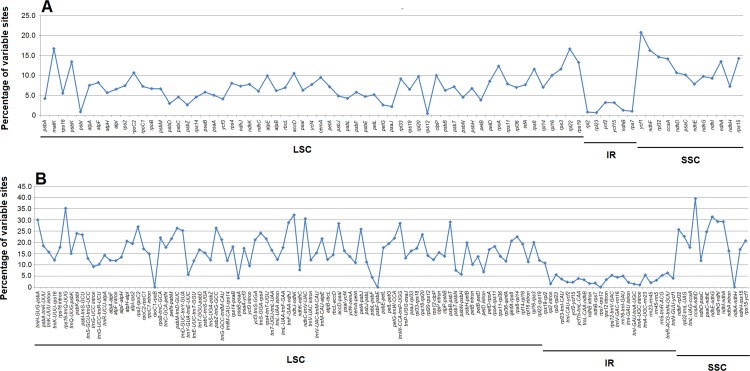
Percentages of variable sites in homologous regions across the six Lamiales chloroplast genomes. (A) coding regions; (B) introns and intergenic spacers (non-coding regions).

The overall sequence identity of the six Lamiales chloroplast genomes was plotted using the software mVISTA with *S*. *dentata* as reference (Fig 2). The results show that the chloroplast genomes within Lamiales are conservative, although divergent regions could be detected. As expected, the IR region is more conserved than the LSC and SSC regions in these species. Meanwhile, non-coding regions reveal a higher divergence than coding regions. In order to check divergent hotspot regions further, all the coding regions and non-coding regions (introns and intergenic spacers) within the six chloroplast genomes were extracted and analysed (S2 Table, Fig 3). The most divergent regions are localized in the intergenic spacers, including *ccsA-ndhD*, *rps16-trnQ-UUG*, *ndhJ-ndhK*, *ndhE-ndhG*, *ndhC-trnV-UAC*, *trnH-GUG-psbA* and *ndhG-ndhI*. These intergenic regions could be used in assessing phylogenetic relationships within Lamiales species. For the coding regions, the most divergent regions are *ycf1*, *matK*, *rpl22*, *rpl32*, *rps15* and *ccsA*. These genes are all located in single copy regions.

**Fig 4:**
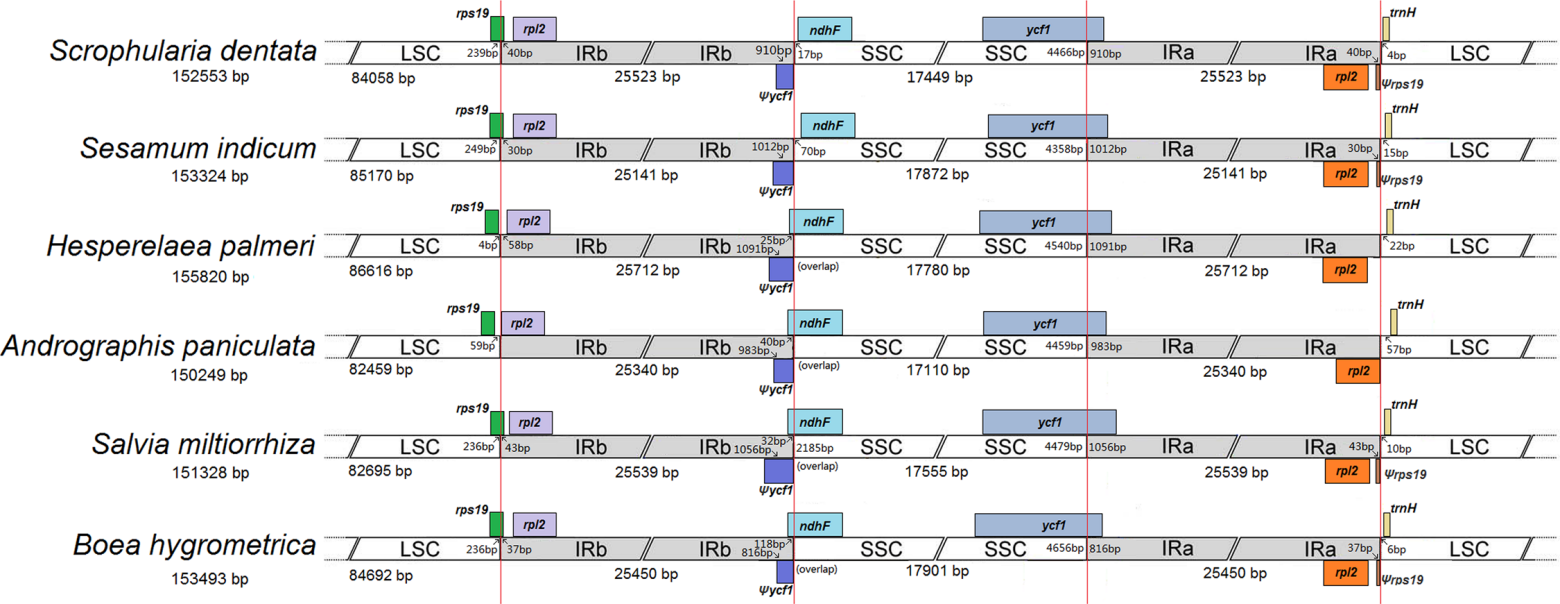
Comparison of the borders of LSC, SSC and IR regions in six Lamiales chloroplast genomes. Ψ indicates a pseudogene. This figure is not to scale.

The expansion and contraction at the borders of IR/SC are common in chloroplast genomes, which is the main reason for the size variation within angiosperm chloroplast genomes [37]. The junctions of LSC/IRb/SSC/IRa of the six Lamiales chloroplast genomes were compared (Fig 4). Although the length of the IR regions of the six species is similar, from 25,141 bp to 25,712 bp, some differences in IR expansions and contractions were observed. The *rps19* genes of *H*. *palmeri* and *A*. *paniculata* are located in the LSC region completely, and the IR region is expanded to *rps19* gene in the other four genomes, with *rps19* pseudogene created at the IRa/LSC border. In *S*. *dentata*, a short *rps19* pseudogene of 40 bp is created. The same pseudogene is 30 bp in *S*. *indicum*, 43 bp in *S*. *miltiorrhiza* and 37 bp in *B*. *hygrometrica*, respectively. The border between IRb and SSC extends into the *ycf1* genes, with *ycf1* pseudogenes created in all of the six species. The length of *ycf1* pseudogene is 910 bp in *S*. *dentata*, 1,012 bp in *S*. *indicum*, 1,091 bp in *H*. *palmeri*, 983 bp in *A*. *paniculata*, 1,056 bp in *S*. *miltiorrhiza* and 816 bp in *B*. *hygrometrica*, respectively. Overlaps are detected between the *ycf1* pseudogene and *ndhF* gene in *H*. *palmeri*, *A*. *paniculata*, *S*. *miltiorrhiza* and *B*. *hygrometrica*, with lengths of 25 bp, 40 bp, 32bp and 118 bp, respectively. The *trnH-GUG* genes are all located in the LSC region, with 4–57 bp apart from the LSC/IRa boundary.

### Repeat and SSR analysis

**Fig 5:**
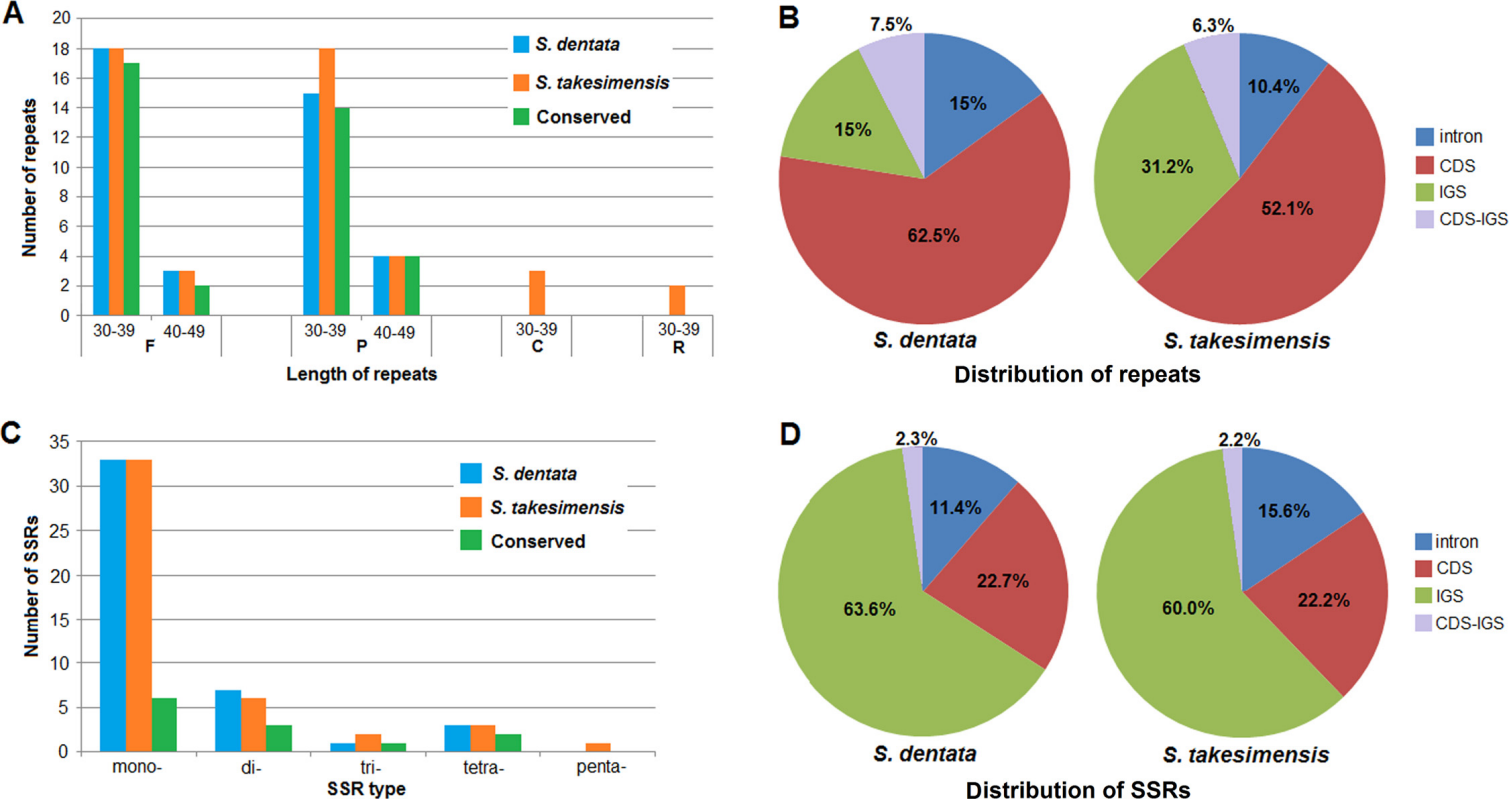
Repeat sequences and simple sequence repeats (SSRs) within *Scrophularia dentata* and *Scrophularia takesimensis* chloroplast genomes. (A) number of different types of repeats. F = forward repeats; P = palindrome repeats; C = complement repeats; R = reverse repeats. (B) distribution of repeats. (C) number of different types of SSRs. (D) distribution of SSRs. CDS = coding sequence; IGS = intergenic spacer; CDS-IGS = part in CDS and part in IGS.

Repeat sequences in the *S*. *dentata* chloroplast genome were analysed by REPuter and the results showed that 21 forward repeats and 19 palindrome repeats were at least 30 bp long per repeat unit with a sequence identity greater than 90% (Table 4). The complement repeats and reverse repeats had not been detected. Of all the repeats found, 33 repeats (82.5%) are 30 to 39 bp long, 7 repeats (17.5%) are 40 to 49 bp long, and the longest repeat is 44 bp. We present a comparative analysis of repeats between *S*.*dentata* and *S*. *takesimensis*, the only one complete chloroplast genome available in Scrophulariaceae [38] (S3 Table, Fig 5A and 5B). Most (92.5%) of the repeats in *S*. *dentata* are conserved and could be found in *S*. *takesimensis*, and there are another 3 complement repeats and 2 reverse repeats found in *S*. *takesimensis*.

**Table 4:** Repeat sequences and their distribution within *Scrophularia dentata* chloroplast genome.

No.	Size(bp)	Type	Repeat 1 start (location)	Repeat 2 start (location)	Region
1	30	F	9597(trnG-UCC*-trnR-UCU)	36418(trnG-GCC*-trnfM-CAU)	LSC
2	30	F	**38641(*psaB*)**	**40865(*psaA*)**	LSC
3	30	F	**43669(*ycf3* intron1)**	**119061(*ndhA* intron)**	LSC; SSC
4	30	F	**88876(*ycf2*)**	**88918(*ycf2*)**	IRb
5	30	F	**91289(*ycf2*)**	**91343(*ycf2*)**	IRb
6	30	F	**91318(*ycf2*)**	**91336(*ycf2*)**	IRb
7	30	F	**145238(*ycf2*)**	**145292(*ycf2*)**	IRa
8	30	F	**145258(*ycf2*)**	**145294(*ycf2*)**	IRa
9	30	F	**147663(*ycf2*)**	**147705(*ycf2*)**	IRa
10	30	F	**43669(*ycf3* intron1)**	**98305(*rps12-trnV-GAC*)**	LSC; IRb
11	31	F	12909(*atpF-atpH*)	69566(*rpl20-rps12*)	LSC
12	32	F	8053(psbI-trnS-GCU*)	35525(psbC-trnS-UGA*)	LSC
13	34	F	**91308(*ycf2*)**	**91326(*ycf2*)**	IRb
14	34	F	**145251(*ycf2*)**	**145269(*ycf2*)**	IRa
15	36	F	**91288(*ycf2*)**	**91306(*ycf2*)**	IRb
16	36	F	**145269(*ycf2*)**	**145287(*ycf2*)**	IRa
17	39	F	**43657(*ycf3* intron1)**	**98293(*rps12-trnV-GAC*)**	LSC; IRb
18	39	F	**43657(*ycf3* intron1)**	**119049(*ndhA* intron)**	LSC; SSC
19	40	F	**91290(*ycf2*)**	**91326(*ycf2*)**	IRb
20	40	F	**145245(*ycf2*)**	**145281(*ycf2*)**	IRa
21	41	F	98291(*trnR-UCU*)	119047(*ndhA* intron)	IRb; SSC
22	30	P	8055(psbI-trnS-GCU*)	**45358(*trnS-GGA*)**	LSC
23	30	P	**43669(*ycf3* intron1)**	**138276(*trnV-GAC-rps12*)**	LSC; IRa
24	30	P	75205(*petB* intron)	75205(*petB* intron)	LSC
25	30	P	35527(psbC-trnS-UGA*)	**45358(*trnS-GGA*)**	LSC
26	30	P	**88876(*ycf2*)**	**147663(*ycf2*)**	IRb; IRa
27	30	P	**88918(*ycf2*)**	**147705(*ycf2*)**	IRb; IRa
28	30	P	**91289(*ycf2*)**	**145238(*ycf2*)**	IRb; IRa
29	30	P	**91318(*ycf2*)**	**145245(*ycf2*)**	IRb; IRa
30	30	P	**91336(*ycf2*)**	**145263(*ycf2*)**	IRb; IRa
31	30	P	**91343(*ycf2*)**	**145292(*ycf2*)**	IRb; IRa
32	34	P	**91308(*ycf2*)**	**145251(*ycf2*)**	IRb; IRa
33	34	P	**91326(*ycf2*)**	**145269(*ycf2*)**	IRb; IRa
34	36	P	**91288(*ycf2*)**	**145269(*ycf2*)**	IRb; IRa
35	36	P	**91306(*ycf2*)**	**145287(*ycf2*)**	IRb; IRa
36	39	P	**43657(*ycf3* intron1)**	**138279(*trnV-GAC-rps12*)**	LSC; IRa
37	40	P	**91290(*ycf2*)**	**145245(*ycf2*)**	IRb; IRa
38	40	P	**91326(*ycf2*)**	**145281(*ycf2*)**	IRb; IRa
39	41	P	**119047(*ndhA* intron)**	**138279(*trnV-GAC-rps12*)**	SSC; IRa
40	44	P	**74375(*psbT-psbN*)**	**74375(*psbT-psbN*)**	LSC

* part in the gene.

Repeat sequences conserved in the *S*. *takesimensis* chloroplast genome are highlighted in bold.

F = forward, P = palindrome, IGS = intergenic spacer.

In general, repeats are mostly distributed in noncoding regions [39, 40]. However, most of the repeats (62.5%) in the *S*. *dentata* chloroplast genome are located in coding regions (CDS), mainly in *ycf2*; and it is similar to that of *S*. *takesimensis* (Fig 5B). Meanwhile, 30% repeats are located in intergenic spacers (IGS) and introns, and 7.5% repeats in parts of the IGS and CDS.

Also, simple sequence repeats (SSRs) exert significant influence on genome rearrangement and recombination [41]. A total of 44 SSRs were detected in the *S*. *dentata* chloroplast genome, accounting for 500 bp of the total sequence (ca.0.33%), and there were 33, 7, 1 and 3 mono-, di-, tri-, and tetra- nucleotide repeats, respectively (Table 5). No penta- or hexa- nucleotide repeats were found. Most of the SSRs are mononucleotide repeats. 43 SSRs (97.7%) are composed of A and T nucleotides, whereas only one is composed of “GTCT” repeat. The high content of AT in SSRs contributes to the AT richness of the chloroplast genome [42, 43]. Among the SSRs, 33 are located in IGS and introns, 10 are found in coding genes, including *ycf1*, *rpoC2*, *atpB*, *rpoA*, *atpA* and *ndhD*, and 1 is located in parts of the IGS and CDS. Compared with *S*. *takesimensis*, 12 SSRs were identity, 30 exhibited length polymorphisms and 2 had not been detected (S4 Table, Fig 5C and 5D). These repeat sequences may be useful in developing lineage-specific markers, which could be widely used in genetic diversity and evolutionary studies of *Scrophularia*.

**Table 5:** Simple sequence repeats within *Scrophularia dentata* chloroplast genome.

Unit	Length	No.	SSR start	SSR-containing region	Region
A	16	1	44297	*ycf3* intron1	LSC
	13	1	12158	*atpF* intron	LSC
	12	2	8024	IGS (*psbI-trnS-GCU*)	LSC
			16175	IGS (*rps2-rpoC2*)	LSC
	11	1	75204	*petB* intron	LSC
	10	8	22732	*rpoC1* intron	LSC
			44557	IGS (*ycf3-trnS-GGA*)	LSC
			59913	IGS (*accD-psaI*)	LSC
			**63314**	**IGS (*petA-psbJ*)**	LSC
			111914	IGS (*ndhF-rpl32*)	SSC
			112458	IGS (*rpl32-trnL-UAG*)	SSC
			137924	IGS (*trnV-GAC-rps12*)	IRa
			152482	IGS (*rpl2-Ψrps19*)	IRa
T	15	1	12921	IGS (*atpF-atpH*)	LSC
	14	1	60099	IGS (*psaI-ycf4*)	LSC
	13	1	69580	IGS (*rpl20-rps12*)	LSC
	12	1	124137	*ycf1*	SSC
	11	8	8632	IGS (*trnS-GCU-trnG-UCC*)	LSC
			14323	IGS (*atpH-atpI*)	LSC
			**18378**	***rpoC2***	LSC
			57534	IGS (*rbcL-accD*)	LSC
			**80092**	**IGS (*infA-rps8*)**	LSC
			80606	IGS (*rps8-rpl14*)	LSC
			112115	IGS (*ndhF-rpl32*)	SSC
			**124952**	***ycf1***	SSC
	10	8	52007	*trnV-UAC* intron	LSC
			**54874**	***atpB***	LSC
			54959	IGS (*atpB-rbcL*)	LSC
			78182	*rpoA*	LSC
			84121	IGS (*rps19-rpl2*)	IRb
			98679	IGS (*rps12 -trnV-GAC*)	IRb
			124603	*ycf1*	SSC
			**124880**	***ycf1***	SSC
AT	12	1	**42432**	**IGS (*psaA-ycf3*)**	LSC
	10	2	**19751**	***rpoC2***	LSC
			13822	IGS (*atpH-atpI*)	LSC
TA	18	1	46524	IGS (*rps4-trnT-UGU*)	LSC
	12	3	**8212**	**IGS (*trnS-GCU-trnG-UCC*)**	LSC
			31456	IGS (*trnE-UUC-trnT-GGU*)	LSC
			42433	IGS (*psaA-ycf3*)	LSC
ATA	12	1	**54928**	IGS (atpB*-rbcL)	LSC
GTCT	12	1	**10993**	***atpA***	LSC
TTTA	12	1	49645	IGS (*ndhJ-ndhK*)	LSC
AATA	12	1	**114611**	***ndhD***	SSC

* part in the gene.

SSRs that are identical in the *S*. *takesimensis* chloroplast genome are highlighted in bold.

## Conclusions

In this paper, we reported the complete sequence of *S*. *dentata* cp genome, as the first whole cp genome in the Sect. Tomiophyllum of the genus *Scrophularia*. This cp genome sequence was compared to other five genomes from the Lamiales species (i.e., *B*. *hygrometrica*, *H*. *palmeri*, *S*. *miltiorrhiza*, *A*. *paniculata* and *S*.*indicum*). No significant structural changes were detected among the chloroplast genomes. All the coding regions and non-coding regions (introns and intergenic spacers) within the six chloroplast genomes were extracted and analysed. The most divergent regions are localized in the intergenic spacers, including *ccsA-ndhD*, *rps16-trnQ-UUG*, *ndhJ-ndhK*, *ndhE-ndhG*, *ndhC-trnV-UAC*, *trnH-GUG-psbA* and *ndhG-ndhI*. For the coding regions, the most divergent regions are *ycf1*, *matK*, *rpl22*, *rpl32*, *rps15* and *ccsA*. These genes are all located in single copy regions. There were some differences in the genome size and IR expansion or contraction; and the divergent regions were analysed. Tandem repeats and SSRs within *S*. *dentata* were compared with those of *S*. *takesimensis*, which may provide markers for the analyses of infraspecific genetic differentiation within *Scrophularia*. In addition, our studies could provide basic data for the alpine medicinal species conservation and molecular phylogenetic researches of Scrophulariaceae and Lamiales.

## Supporting Information

S1 Table List of all pairs of primers used for assembly validation. (DOC)

S2 Table Percentages of variable sites across the six Lamiales chloroplast genomes. (XLS)

S3 Table Repeat sequences within *Scrophularia takesimensis* chloroplast genome. (XLS)

S4 Table Simple sequence repeats within *Scrophularia takesimensis* chloroplast genome. (XLS)

## Abbreviations

cp: chloroplast
LSC: large single-copy region
SSC: small single-copy region
IR: inverted repeat
bp: base pair(s)
rRNA: ribosomal RNA
tRNA: transfer RNA
IGS: intergenic spacer
CDS: coding sequence
SSR: simple sequence repeat
