# Supplementary material for: The Complete Chloroplast Genome of Ye-Xing-Ba (Scrophularia dentata; Scrophulariaceae), an Alpine Tibetan Herb
Source: PLoS One. 2016 Jul 8;11(7):e0158488. doi: 10.1371/journal.pone.0158488 (PMC4938499; doi:10.1371/journal.pone.0158488)
Supplement: S1 Table — (DOC) [file pone.0158488.s001.doc]

**Table S1.**

. List of all pairs of primers used for genome validation.

| Regions | Sequence (5’>3’) |
| --- | --- |
| Junction of LSC/IRb | F ACTTGCGTTGACTTCTGCTTTACT |
|  | R GGGGGAGGTCCGTTTGATA |
| Junction of IRb/SSC | F ATGGCTTGGATTGGTATTA |
|  | R GAAAAAGGGAGGGTCTATT |
| Junction of SSC/IRa | F TTACTTTGGGGGTTTTTAGGATA |
|  | R GGTAGCCAGATTAGTCAACATTTA |
| Junction of IRa/LSC | F GGTAAGCGTCCTGTAGTAAGA |
|  | R TAAAATAAAGGAGCAATAACG |
| *psbA-trnH* | F GTTATGCATGAACGTAATGCTC |
|  | R CGCGCATGGTGGATTCACAATCC |
| *rbcL* | F ATGTCACCACAAACAGAAAC |
|  | R TCGCATGTACCTGCAGTAGC |
| *matK* | F CGTACAGTACTTTTGTGTTTACGAG |
|  | R ACCCAGTCCATCTGGAAATCTTGGTTC |
| *rpoC1* | F GGCAAAGAGGGAAGATTTCG |
|  | R CCATAAGCATATCTTGAGTTGG |
| *trnL(UAA)* | F CGAAATCGGTAGACGCTACG |
|  | R GGGGATAGAGGGGACTTGAAC |
| *trnL(UAA)-trnF(GAA)* | F GGTTCAAGTCCCTCTATCCCC |
|  | R GGTTCAAGTCCCTCTATCCCC |
| *trnS(GCU)-trnG(UCC)* | F GCCGCTTTAGTCCACTCAGC |
|  | R GAACGAATCACACTTTTACCAC |
| *rpl20-rps12* | F TTTGTTCTACGTCTCCGAGC |
|  | R GTCGAGGAACATGTACTAGG |
| *atpB-rbcL* | F GAAGTAGTAGGATTGATTCTC |
|  | R GAAGTAGTAGGATTGATTCTC |
